# Strategies and challenges in containing antimicrobial resistance in East Africa: a focus on laboratory-based surveillance

**DOI:** 10.1186/s13756-025-01662-y

**Published:** 2025-11-07

**Authors:** Andrea Molina, Théogène Ihorimbere, Néhémie Nzoyikorera, Eunice Jennifer Nambozo, Saudah Namubiru, Susan Nabadda, Godfrey Pimundu, Susan Mahuro Githii, Gwokpan Awin Nykwe, Abe G. Abias, Felician L. Msigwa, Reuben Ndagula, Nyambura Moremi, Flora Rwanyagatare, Josiane Tuyishimire, Therese Mukankwiro, Noel Gahamanyi, Isabelle Mukagatare, Maike Lamshöft, Julien A. Nguinkal, Emmanuel Achol, Hakim I. Lagu, Eric Nzeyimana, Jürgen May, Florian Gehre, Muna Affara

**Affiliations:** 1 Department Infectious Disease Epidemiology, Bernhard Nocht Institute for Tropical Medicine, 20359 Hamburg, Germany; 2 National Reference Laboratory, Institut National de Santé Publique, Ministry of Health and Fight against AIDS, Bujumbura, Burundi; 3 Central Public Health Laboratories, National Health Laboratories, Ministry of Health, Kampala, Uganda; 4 National Public Health Laboratory, Ministry of Health, Nairobi, Kenya; 5 National Public Health Laboratory, Ministry of Health, Juba, Republic of South Sudan; 6 National Public Health Laboratory, Ministry of Health, Dar es Salam, Tanzania; 7 Biomedical Services Department, Biomedical Centre, Kigali, Rwanda; 8 Health Department, East African Community (EAC), Arusha, Tanzania; 9 German Center for Infection Research (DZIF), Hamburg-Borstel-Luebeck- Riems, Germany; 10 Tropical Medicine II, University Medical Center Hamburg-Eppendorf (UKE), 20251 Hamburg, Germany; 11 Escuela de Zootecnia, Facultad de Ciencias Agroalimentarias, Universidad de Costa Rica, San José, 11501-2060 Costa Rica

**Keywords:** Antimicrobial resistance, AMR surveillance, East african community (EAC), AMR national action plan, One health

## Abstract

**Background:**

Antimicrobial resistance (AMR) is increasing worldwide, undermining strides in public health and the economy, particularly in low- and middle-income countries. Africa is the continent with the highest death rate attributed to antimicrobial-resistant infections. There is a lack of information on AMR mitigation strategies and their implementation in the region. The aim of this study was to analyze national strategies to tackle AMR with focus on AMR surveillance in the East African Community (EAC) and their implementation status including the analysis of strengths, weaknesses, opportunities, and threats.

**Methods:**

Within our expert group (composed of representatives from the National Public Health Laboratories (NPHL), Ministries of Health of Burundi, Kenya, Rwanda, South Sudan, Tanzania, and Uganda) we used a qualitative approach to analyze AMR National Action Plans (NAPs), AMR surveillance programs, publications and reports on the AMR situation and strategies in the EAC. Results: We found varying levels of implementation of antimicrobial resistance (AMR) strategies among East African Community (EAC) Partner States. For example, progress in key steps for the sustainable implementation of National Action Plans on AMR (AMR-NAPs) ranged from 7% in Burundi to 94% in Kenya. The overall accomplishment of the WHO checklist for AMR surveillance also varied: 44% in South Sudan, 61% in Burundi, 89% in Rwanda, 94% in Tanzania, and 100% in both Uganda and Kenya. Within EAC Partner States, the detection of bacterial pathogens and their antimicrobial susceptibility profiles is coordinated by national reference laboratories. Most EAC countries have established AMR surveillance systems. However, challenges such as limited laboratory testing capacity, low representativeness of surveillance data, lack of integration among existing systems, and financial constraints undermine efforts to curb AMR.

**Conclusions:**

Regional collaboration among EAC Partner States is essential for an effective and sustainable response to antimicrobial resistance. Strengthening joint efforts will enable countries to share resources, harmonize surveillance systems, and address common challenges more efficiently. The EAC Regional Network of Reference Laboratories is one example of a regional mechanism that can support such collaboration. The findings of this study will inform the development of a regional AMR strategy focused on laboratory-based surveillance and help guide the prioritization of technical and financial support across the EAC region.

**Supplementary Information:**

The online version contains supplementary material available at 10.1186/s13756-025-01662-y.

## Background

The increase in antimicrobial resistance (AMR) is a problem that undermines public health and economic development worldwide, particularly in low- and middle-income countries (LMICs) [1]. AMR is the ability of microbes to nullify the effects of drugs, which become ineffective and lead to infections that are difficult to treat [2, 3].

In 2019, AMR was associated with 5 million deaths worldwide, with Africa among the most affected regions reporting 24 deaths per 100,000 people attributable to AMR [1, 4]. In addition, national and regional surveillance reports warn about the high prevalence of AMR in pathogenic bacteria in East Africa [4–7]. Possible reasons for the increased prevalence of AMR pathogens, alongside the overall high load of infectious diseases in the region, include limited diagnostic capacity and AMR surveillance systems, as well as unregulated use of antibiotics, poor water, sanitation, and hygiene (WASH), lack of advanced research in antibiotic development, and financial constraints [2, 3].

Global and national efforts have been made to counteract the AMR crisis. At the 2015 World Health Assembly, the United Nations (UN) Member States endorsed the Global Action Plan to address the growing problem of AMR. Each UN Member State agreed to develop a National Action Plan (NAP) on AMR aligned with the Global Action Plan and to implement relevant policies to prevent, control, and monitor AMR, considering national and regional priorities [8]. As part of the development and implementation of the AMR-NAPs [9, 10], East African countries have worked on developing national AMR surveillance programs [8], adopting the One Health approach to curbing AMR [11, 12], education and awareness campaigns [13], improving access to vaccines and the use of alternative treatment options [14].

Furthermore, key frameworks and guidance documents have been developed to address AMR in Africa, such as the Africa CDC Framework for Antimicrobial Resistance, which aims to guide efforts to measure, prevent, and mitigate harms from AMR organisms in the region [15]; and the Antimicrobial Resistance Surveillance Guidance for the African Region, which aims to tackle the complexities of AMR surveillance [16]. Despite such efforts, the East African region faces significant challenges that need to be addressed to counteract this global crisis [17, 18]. According to the WHO TRACSS evaluation, the majority of East African countries have not yet formalized a multisectoral coordination mechanism to address antimicrobial resistance (AMR). This mechanism should include the human, animal, and environmental health sectors, which are all essential for effectively tackling the AMR crisis. [11]. Moreover, the main challenges faced by East Africa include limited information on the status of AMR, low AMR laboratory detection capacity [19] and scarcity of studies assessing AMR mitigation strategies in the region [20, 21]. The East African Community is a regional intergovernmental organization of eight Partner States: the Republic of Burundi, the Republic of Kenya, the Republic of Rwanda, the Republic of South Sudan, the United Republic of Tanzania, the Republic of Uganda, the Democratic Republic of the Congo (DRC), and the Federal Government of Somalia. The latter two are the most recent members and joined in July 2022 and December 2023, respectively.

The EAC, in collaboration with the Bernhard Nocht Institute for Tropical Medicine (BNITM), is implementing the EAC mobile laboratories project “EAC Regional Network of Public Health Reference Laboratories for Communicable Diseases”, which aims to establish a sustainable laboratory infrastructure and response network across the East African Community and to strengthen AMR surveillance. Within this project, we intend to develop a harmonized, regional laboratory-based AMR surveillance strategy to be implemented in all EAC Partner States. To achieve this goal, we conducted a baseline assessment of national and regional efforts to contain AMR in the EAC, with a particular focus on laboratory-based surveillance using a qualitative approach and a regional strengths, weaknesses, opportunities, and threats (SWOT) analysis for AMR containment in the EAC to guide the prioritization of technical and financial support.

## Methods

### Study design

This research used a qualitative approach to describe the strategies used to contain AMR in six EAC Partner States: Burundi, Kenya, Rwanda, South Sudan, Tanzania, and Uganda. We conducted expert group discussions in 2023 in Kenya and reviewed policy documents, articles, and global and national reports on AMR-containing measures, including the implementation of AMR-NAPs, the laboratory capacity to detect antibiotic resistance and AMR surveillance and stewardship programs in the EAC Partner States.

### Expert group composition and data collection

In this study, a qualitative, narrative research design was used. The design was chosen to achieve the exploratory aim of gaining insights from key players in the region. The National Public Health Laboratories coordinate national laboratory AMR surveillance activities and are involved in development and implementation of AMR-NAPs in each country. Purposive sampling was used to select eight participants who are senior experts and have a clinical microbiology background. The National Public Health Laboratories/Ministries of Health from Burundi, Kenya, Rwanda, South Sudan, Tanzania, and Uganda were approached to nominate experts involved in national laboratory-based AMR surveillance and play an active role in the interdisciplinary and intersectoral development of the AMR-NAPs. The expert group discussion was facilitated during a workshop and covered the following four themes: (i) the national policy to contain AMR, including its level of implementation; (ii) AMR laboratory-based surveillance programs, including laboratory capacity and quality control programs; (iii) AMS programs, including awareness campaigns; and (iv) the gaps and challenges in containing AMR in EAC, including a SWOT analysis. Participants were guided by a question catalogue on the above-mentioned topics, and were asked to elaborate on their countries’ policies verbally and in written statements. Information was documented during the discussion. Additionally, to measure the status of accomplishment of key requisites for the development and operationalization of the AMR-NAPs and the status of the AMR surveillance programs, two established instruments were used among the participants: [1] the Summary checklist for six steps for sustainable implementation of NAPs on AMR from the WHO Implementation Handbook for NAPs on AMR [22], and the WHO National antimicrobial resistance surveillance systems and participation in the Global Antimicrobial Resistance Surveillance System (GLASS): core components checklist and questionnaire [23].

### Data analysis

Thematic analysis was conducted based on four previously identified themes. This analysis synthesized information from expert group discussion transcripts, completed checklists and questionnaires, as well as relevant policy documents, academic articles, and global and national reports on strategies to contain AMR in the EAC. Information was extracted systematically and grouped by country. As a first step, all documents were reviewed, and the information classified under each of the thematic areas. The analysis followed the six stages of thematic content analysis in qualitative research [24].

**Fig. 1:**
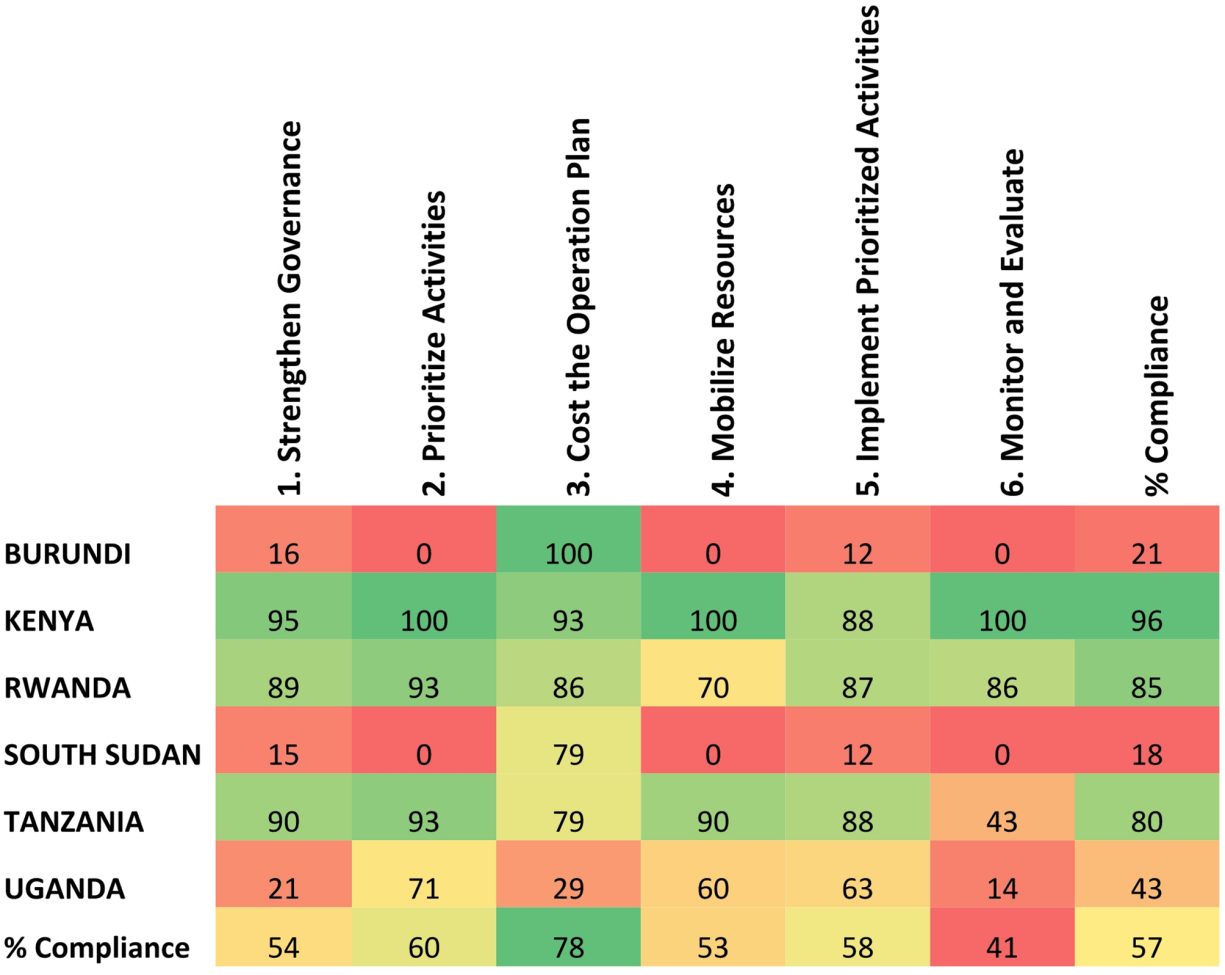
Country-NPHL self-assessment of accomplishment of the six WHO steps for the sustainable implementation of AMR-NAPs. The percentage of accomplishment for each step of the WHO implementation handbook for NAPs on AMR [22] is reported on a scale of 0 (noncompliance [red] to 100 (full compliance [green])

To improve the reliability of the narrative data collected, and to calculate an accomplishment score, a self-assessment was conducted using the Annex 9: “Summary checklist for six steps for sustainable implementation of NAPs on AMR,” as provided in the WHO Implementation Handbook for National Action Plans on Antimicrobial Resistance [22]. Each country representative evaluated the accomplishment of the following six key steps for sustainable NAP implementation: (i) establishing/strengthening multisectoral coordination, collaboration, and governance for the implementation of the NAP for AMR, (ii) prioritizing activities for implementation, (iii) developing cost-related operational plans, (iv) identifying funding gaps and mobilizing resources for implementation, (v) implementing NAP AMR activities, and (vi) monitoring and evaluating the NAP for AMR. For each accomplished step, 8–19 action points were awarded (Supplementary material 1) and a score was created using the formula: Score = (Number of action points accomplished/Total number of action points) * 100. The results per country are shown in Fig. 1.

To specifically assess the status of the laboratory-based AMR surveillance program in the EAC Partner States, the document “National antimicrobial resistance surveillance systems and participation in the Global Antimicrobial Resistance Surveillance System (GLASS) Core components checklist and questionnaire” [23] was used. Each representative completed this checklist, which consists of 18 requirements for AMR surveillance programs. These requirements are shown in Table 2. We calculated a score by dividing the number of requirements met by each country by the total number of requirements.

## Results

The results are presented following the four themes of the thematic analysis: (i) the national policy to contain AMR, including its level of implementation; (ii) AMR laboratory-based surveillance programs, including laboratory capacity and quality control programs; (iii) AMS programs, including awareness campaigns; and (iv) the gaps and challenges in containing AMR in EAC, including a SWOT analysis.

### National policy to contain AMR

All Partner States of the East African Community developed a NAP for the prevention and containment of AMR. These NAPs are aligned with the One Health approach, incorporating coordinated actions across the human health, animal health, and environmental sectors [25–30]. The NAPs are based on the five strategic objectives outlined in the Global Action Plan on AMR [31]:

- To improve awareness and understanding of antimicrobial resistance.
- To strengthen knowledge through surveillance and research.
- Reducing the incidence of infection.
- To optimize the use of antimicrobial agents.
- To ensure sustainable investment in countering antimicrobial resistance.

In addition to the five core objectives, Tanzania and Kenya have included “Strengthen coordination, collaboration, and governance” as a sixth strategic objective—listed as the first priority in their second-generation NAPs. This addition underscores the importance of intersectoral coordination and joint action in effectively responding to AMR [28, 30].

The results of the self-assessment of the accomplishment of the six WHO steps for the implementation of AMR-NAPs, show that **Kenya**, **Rwanda and Tanzania** have completed more than 80% of all the key requisites to develop and operationalize their AMR-NAPs (Fig. 1) (Supplementary Table). In **Uganda**, the integration of the different sectors and ministries of the One Health NAP is still in progress, and some of the Technical Working Groups for AMR-NAP implementation are not fully functional. In **Burundi**, the AMR-NAP was launched in April 2020. However, the steps for AMR-NAP implementation are still at an early stage (Fig. 1). **South Sudan** developed its first National Action Plan on Antimicrobial Resistance (AMR-NAP) in 2023, which is set to be launched in 2025. The plan includes a detailed budget, a monitoring and evaluation framework, and an implementation matrix. It features a strong multi-sectoral coordination mechanism involving relevant ministries and has received political endorsement from all relevant ministries and the Ministry of Cabinet Affairs.

From a regional perspective, most progress has occurred in the areas of governance (step 1) and prioritizing activities (step 2). In step 1, all countries have established a multisectoral AMR committee that includes at least the human and animal health sectors and has a designated AMR focal person. To prioritize NAP activities (step 2), all EAC countries have conducted a national assessment of the current AMR situation, including the information submitted to the Global Database for Tracking AMR Country Self-Assessment Survey (TrACSS) [11].

Although many of the steps included in the resource mobilization component (Step 4), such as identifying existing and potential funders, have already been taken, the sustainability of the financial resources for AMR-NAP implementation remains a challenge. In general, the highest number of pending actions was found in steps 4 (mobilizing resources to support the activities included in the AMR-NAP) and 6 (monitoring and evaluating the progress in NAP implementation) (Fig. 1). Furthermore, in this self-assessment, the countries highlighted the need to strengthen their capacity in AMR surveillance, awareness, infection prevention, and optimization of antimicrobial use.

In addition to these six steps recommended by the WHO, we analyzed, within the expert group discussions, the implementation of planned activities on AMR according to National Action Plans. Furthermore, we compared the narratives provided by the experts with the official 2022 TrACSS [11] country responses, where the same topics were covered. **Kenya** and **Tanzania** have implemented actions concerning all five objectives of the AMR Global Action Plan mentioned above. In **Tanzania**, emphasis has been given to implementation of actions that encourage regulatory authorities to control the quality, distribution, and use of antibiotics in humans, animals, and agriculture. Additionally, antibiotic stewardship in Tanzania includes monitoring and evaluating the use and consumption of antibiotics in health facilities [32]. In **Uganda**, actions have been taken particularly in antibiotic resistance surveillance, antibiotic stewardship, and infection prevention and control corresponding to the first three objectives of the Global Action Plan [17, 33].

In **Rwanda**, the One Health NAP on AMR was adopted in June 2020. The main priorities in NAP implementation have been to improve the laboratory capacity to conduct AMR testing for AMR surveillance and to raise awareness [34, 35].

In **Burundi**, since approval of the NAP in 2020, few planned activities have been conducted, including the nomination of an AMR focal person and a multisectoral committee, the designation of sentinel sites for surveillance of antibiotic resistance and development of standard operating procedures for antibiotic sensitivity testing.

In **South Sudan**, antimicrobial resistance has not been a public priority, in part owing to nascent infection disease surveillance and limited laboratory capacity of the country. In 2023, the Ministry of Health, supported by WHO, FAO and Africa CDC, established an AMR multisector coordination committee and developed its first AMR-NAP, which is expected to be launched in 2025.

These statements from the expert group are consistent with findings from the 2022 TrACSS report [11]. According to the report, Kenya and Tanzania have achieved nationwide implementation for all 5 indicators of their NAPs and a sustained capacity in optimizing antimicrobials use [11]. Uganda has accomplished a “developed” level of progress in the TRACSS indicators related to objectives 1–3 of its AMR-NAP [11]. Rwanda has reached a “developed” level of progress in the national AMR surveillance in the human sector. In contrast, Burundi and South Sudan presented a limited progress across most TrACSS indicators for the human health sector [11], which aligns with narrative data from the expert group discussions. The expert group in alignment with the TrACSS self-assessment for Rwanda, Burundi and South Sudan found the monitoring of antimicrobial consumption [11] to be a main limitation.

In general, according to our expert group, little progress has been made in Objectives 4 (optimizing the use of antimicrobial agents) and 5 (ensuring sustainable investment) of the NAPs on AMR in all EAC Partner States. The main challenges faced by countries during the implementation of their AMR-NAPs were identified by expert group discussion as follows: (i) lack of stable financial resources to support planned activities, (ii) inadequate enforcement of antimicrobial use regulations, (iii) staffing shortages and (iv) disruption of planned activities during the COVID-19 pandemic. In addition, the lack of joint work and coordination between the human health, animal health and environmental sectors is a problem that limits the effective application of AMR-NAPs [32, 33].

Tanzania has already launched its second AMR National Action Plan (NAP) for the period 2023–2028, and Kenya has released its second NAP covering the period 2023–2027. Uganda is in the final stages of preparing its second AMR-NAP. A key focus of these second-generation plans is to improve the costing of implementation, mobilize the necessary resources, and strengthen multisectoral coordination mechanisms across all sectors, in line with the One Health approach.

### Laboratory-based AMR surveillance programs

In the EAC region, the level of development and coverage of the AMR national surveillance systems varies greatly among EAC Partner States (Table 2). In the case of **Burundi**,** Kenya**,** Rwanda**,** Tanzania and Uganda**, there is a national AMR surveillance system in place that collects data on antibiotic resistance in bacterial infections in healthcare facilities, as well as an established network of surveillance sites (Table 1), a designated national reference laboratory for AMR, and a national coordination center that produces AMR reports [6, 30, 33, 36] (Table 2). These national surveillance programs consider GLASS priority pathogens *Acinetobacter* spp., *E. coli*, *Klebsiella pneumoniae*, *Neisseria gonorrhoeae*, *Salmonella* spp., *Shigella* spp., *Staphylococcus aureus*, and *Streptococcus pneumoniae* and the priority clinical specimen types [37]. However, not all the recommended pathogens and specimen types have been incorporated into national surveillance programs in all countries (Table 1). In addition to the GLASS pathogens, some countries’ surveillance programs also cover pathogens of regional relevance, such as *Vibrio cholerae* in stool and *Neisseria meningitidis* in cerebrospinal fluid (CSF), due to their high prevalence in Uganda; *Pseudomonas aeruginosa* and *Enterococcus faecalis*, due to their association with nosocomial infections in Kenya, Rwanda and Uganda (Table 1).

**Table 1:** Characteristics of the National AMR surveillance system in the EAC

Country	Number of surveillance sites	Period of surveillance	Sample types for AMR surveillance	Microorganisms for surveillance
Burundi	9	Since 2020	Urine	*Escherichia coli* *Klebsiella pneumoniae*
Kenya	21	Since 2016	Stool	*Streptococcus pneumoniae*
			Urine	*Pseudomonas aeruginosa*
			Blood	*Escherichia coli*
			Wounds swabs	*Acinetobacter baumannii*
			urethral and cervical swabs	*Salmonella* spp
			CSF	*Shigella* spp
				*Klebsiella pneumoniae*
				*Staphylococcus aureus*
Rwanda	12	Since 2020	Stool Urine	*Escherichia coli*
			Blood	*Salmonella* spp
			Wounds swabs/pus	
			Cerebrospinal fluid (CSF)	*Shigella* spp
				*Klebsiella pneumoniae* *Acinetobacter baumannii* *Staphylococcus aureus* *Neisseria meningitidis* *Pseudomonas aeruginosa* *Streptococcus pneumoniae*
Tanzania	9	Since 2020	Urine	*Escherichia coli*
			Blood	*Acinetobacter spp*
				*Salmonella* spp.
				*Klebsiella pneumoniae*
				*Streptococcus pneumoniae*
Uganda	22	Since 2016	Stool	*Escherichia coli*
			Urine	*Klebsiella pneumoniae*
			Blood	*Staphylococcus aureus*
			urethral and cervical swabs	*Acinetobacter baumannii*
			Cerebrospinal fluid (CSF)	*Salmonella spp.*
				*Shigella spp.*
				*Neisseria gonorrhoeae*
				*Streptococcus pneumoniae*
				*Vibrio cholerae*
				*Pseudomonas aeruginosa*
				*Enterococcus faecalis*

Although all EAC countries are included in GLASS, only Uganda, Kenya and Tanzania regularly report data from their AMR surveillance programs (Table 2). Presently, Tanzania reports only AMR data from blood and urine samples (Table 1) [38]. In **South Sudan**, the AMR surveillance program is in the planning stage, hence, there are currently no active surveillance sites.

Most bacterial identification and antibiotic susceptibility testing (AST) in EAC Partner States are performed manually, and access to high-tech automated equipment is mostly limited to National Reference Laboratories (NRLs). The use of automated technologies in identification and ASTs is limited by the availability and increased costs of reagents and consumables. AMR data are obtained mainly from hospitals and NRLs and are reported manually or via different laboratory databases, such as WHONET.

**Table 2:** Country-NPHL self-assessment of compliance with WHO requirements for AMR surveillance programs

	South Sudan	Burundi	Rwanda	Tanzania	Uganda	Kenya
Ongoing AMR surveillance in humans		✓	✓	✓	✓	✓
National AMR report				✓	✓	✓
NAP in place	✓	✓	✓	✓	✓	✓
AMR surveillance included in NAP	✓	✓	✓	✓	✓	✓
Existing AMR national coordinating center (NCC)	✓	✓	✓	✓	✓	✓
NCC with defined functions	✓		✓	✓	✓	✓
National reference Lab for AMR surveillance	✓	✓	✓	✓	✓	✓
AMR surveillance include all WHO priority specimens			✓		✓	✓
Priority pathogen-antimicrobial combination defined	✓		✓	✓	✓	✓
Quality control of surveillance sites			✓	✓	✓	✓
National surveillance system use a single guideline for AST	✓	✓	✓	✓	✓	✓
Surveillance site with GLASS requirements			✓	✓	✓	✓
Capacity to collect and report good-quality data		✓	✓	✓	✓	✓
Surveillance sites capable to identify pathogen and AMR		✓	✓	✓	✓	✓
Laboratory quality control system		✓	✓	✓	✓	✓
Laboratory has data management capacity		✓	✓	✓	✓	✓
Country enrolled in GLASS	✓	✓	✓	✓	✓	✓
Country sending AMR data to GLASS				✓	✓	✓
% Compliance	**44**	**61**	**89**	**94**	**100**	**100**

Countries are ranked in ascending order by their overall percentage of accomplishment of the WHO checklist for AMR surveillance programs [23, 37].

In most EAC Partner States, the national reference laboratory has the necessary infrastructure to identify bacterial pathogens and determine antibiotic susceptibility profiles. However, in district hospitals and some NRLs, as is the case in Burundi and South Sudan, laboratory diagnosis of AMR is limited by the availability of reagents and consumables; in particular, the analysis of blood samples is affected by the lack of the necessary culture media.

In the EAC, **Uganda**,** Kenya** [39], **Rwanda and Tanzania** have implemented laboratory quality management systems, including a national microbiology external quality assessment (EQA) program, and the reference laboratories are part of international accreditation programs. However, not all clinical microbiology laboratories in these countries actively participate in standard quality control programs. In **Burundi and South Sudan**, bacteriological laboratories do not perform quality control tests on a regular basis, and the application of quality control measures is restricted by a lack of resources. Despite country constraints in maintaining quality control systems, all national reference laboratories have standard operating procedures (SOPs) for the identification of bacterial pathogens and antibiotic resistance profiles on the basis of international standardized guidelines such as the Clinical Laboratory Standards Institute (CLSI) or the European Committee on Antimicrobial Susceptibility Testing (EUCAST).

The laboratory-based AMR surveillance in the EAC Partner States is financed through the national budget and donors. The most important funders of AMR surveillance actions in the region are the Fleming Fund, World Bank, ASLM, WHO, and CDC. Most of this support is neither consistent nor uniform across the EAC region. Typically, support is linked to short- or medium-term projects, which directly impact the laboratories’ capacity to conduct antimicrobial resistance (AMR) detection analyses. The exact proportion of activities funded by national governments versus those funded by external entities varies among EAC countries and also fluctuates significantly over time. What is consistent across all countries in this study is a strong dependence on external funding to sustain AMR surveillance programs.

The most crucial challenges regarding laboratory-based AMR surveillance found in expert group discussions include the stockouts of laboratory diagnostic materials, irregularities in equipment maintenance, lack of integrated reporting systems, high turnover of trained laboratory personnel, limitations in well-designed laboratory infrastructure and low clinician demand for bacteriological and antibiotic susceptibility testing services.

### Antimicrobial stewardship (AMS)

The implementation of stewardship programs is an important part of the AMR Global Action Plan [8]. In **Burundi** and **South Sudan**, stewardship programs are part of the planned activities, even though the implementation of these programs is at a very early stage [29]. In Burundi, for example, this has included three sensitization sessions for doctors on the rational use of antibiotics and the importance of the laboratory in performing diagnostic tests, training technicians in the detection of bacterial pathogens and mentoring personnel at AMR surveillance sites. In South Sudan, AMS programs include the development of local guidelines for prescription of antibiotics in hospitals [40]. **Rwanda** developed national AMS guidelines for health care settings, the main purpose of which is to provide guidance and direction to health care workers and institutions in Rwanda on how to operationalize AMS in health care settings and the community at large to promote optimal use of antimicrobial agents. The country selected twelve [12] sentinel sites (hospitals) for effective implementation of antimicrobial stewardship and provided stewardship training to AMS committees of selected sites. In **Kenya**, antimicrobial stewardship programs have been established in 15 public and major private hospitals. Furthermore, assessments of stewardship activities have been conducted, and a multisectoral AMR training curriculum including AMS and awareness modules has been developed [41]. In **Uganda**, in addition to AMS and awareness activities in health care centers, there are community education campaigns about appropriate antimicrobial use. **Tanzania** has developed standard treatment guidelines (STGs) and a National Essential Medicine List (NEMLIT) to guide antimicrobial prescription in health facilities and AMR awareness and education activities [42].

With respect to antimicrobial use surveillance, most EAC countries do not measure and/or report country-wide antibiotic use to national or global systems such as GLASS.

### Gaps and challenges for containment of AMR in EAC

We analyzed strengths, weaknesses, opportunities, and threats of the EAC Partner States to contain AMR in the region. For this analysis, we considered expert group discussions, the AMR-NAP of each country, and reports on the AMR situation in Africa, such as reports from the Mapping Antimicrobial Resistance and Antimicrobial Use Partnership (MAAP) and the African Society for Laboratory Medicine (ASLM) [20, 21].

As identified through the SWOT analysis (Table 3), expert discussions highlighted that one of the main **strengths** of the EAC Partner States lies in the perceived existence of strong collaborative relationships between the Ministries of Health, particularly through the EAC Regional Network of Reference Laboratories. These relationships are supported by shared initiatives, joint training programs, collaborative commissions and ongoing information exchange.

**Table 3:**
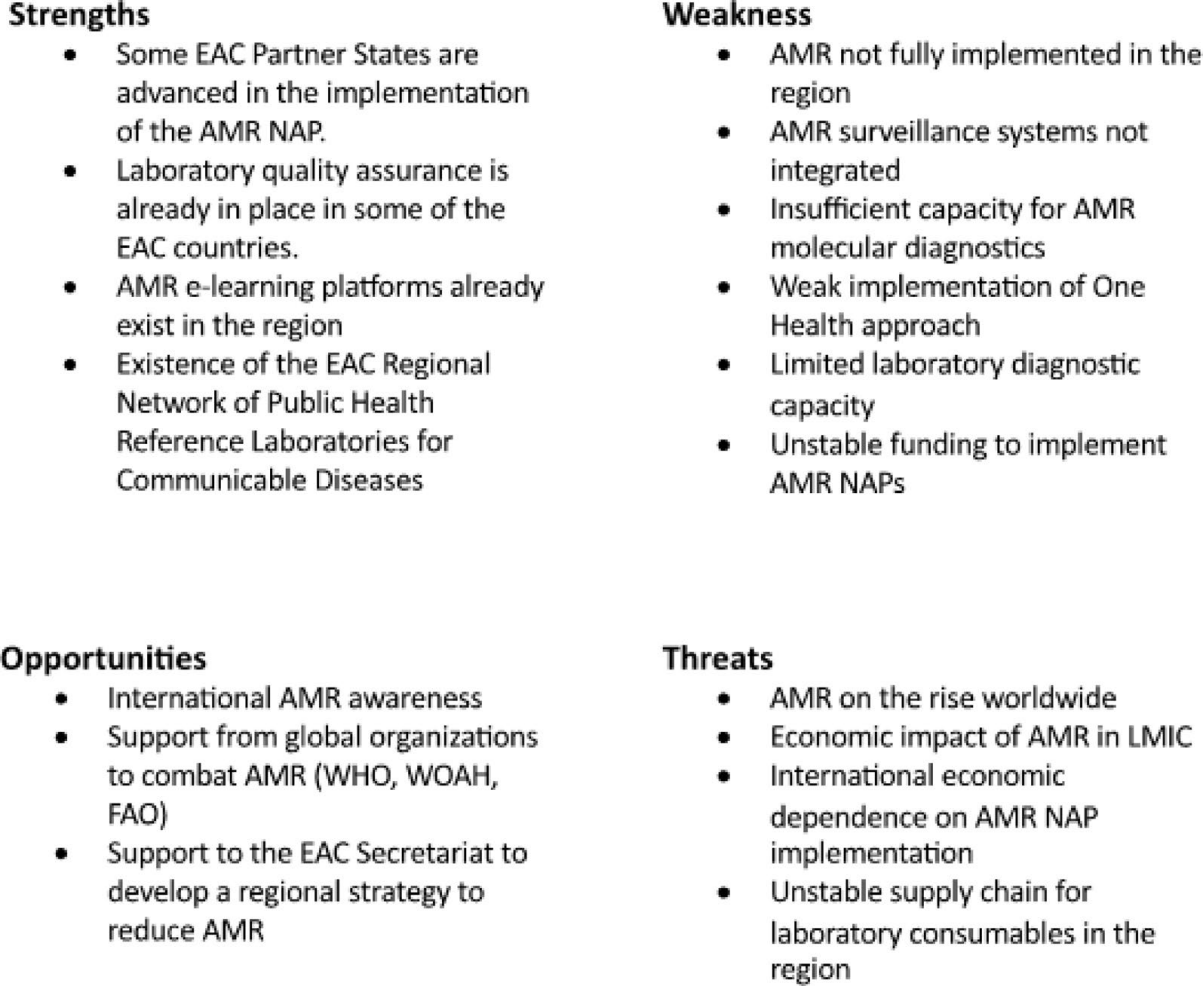
Characteristics of the National AMR surveillance system in the EAC

Among the **strengths** found in the SWOT analysis in different countries across this network are laboratory-based AMR surveillance programs, quality control systems for laboratory analyses, and online AMR-education tools. While countries have different national priorities, this diversity offers an **opportunity** for mutual learning and complementarity. The network can serve as a platform to pool efforts, achievements, and lessons learned, enabling countries to adapt and scale up successful interventions at the regional level. Converging national efforts into coordinated regional actions could help address the **common weaknesses** founded in the SWOT analysis, such as limited laboratory capacity, low surveillance coverage, lack of system integration, and financial constraints (Table 3).

The SWOT analysis identified several **regional threats**, including international economic dependence on donors to conduct AMR containing activities, an unstable supply chain for laboratory consumables for AMR testing, and the ongoing rise of AMR worldwide. Despite these challenges, there are also significant **opportunities**. The expanded regional network can capitalize on the increasing global awareness of the AMR crisis and the support from regional and international organizations—such as the EAC, WHO, the Food and Agriculture Organization (FAO), and the World Organization for Animal Health (WOAH)—to enhance coordinated actions against AMR.

## Discussion

The results of this study are highly relevant, as they identify not only the main interventions that have been introduced to curb AMR in East Africa, a region severely affected by resistant infectious diseases, but also their level of implementation and limitations. Importantly, the experts who participated in the group discussions were front-row specialists and were well informed about the national strategies used to contain AMR in their countries. One of the main limitations of this study is that it focuses on AMR control measures implemented or coordinated by government agencies at the national level. Therefore, initiatives developed solely at the local or private level may not be included in this analysis. Additionally, experts who took part in the discussions are government employees prone to having conflicts of interest, thereby overestimating the accomplishments of their respective governments. However, this risk of bias was minimized by working together over a prolonged time, with established transparency and trust among the participants. Qualitative research always harbors a certain level of interpretation involved in narratives, therefore we used checklists, national and global reports to verify the reporting.

All EAC Partner States have developed a National Action Plan on Antimicrobial Resistance (AMR-NAP) and established a National AMR Committee to coordinate strategies and activities to contain AMR at the national level. However, progress in implementing key steps for the sustainable execution of AMR-NAPs varies considerably among countries. While some have advanced significantly, others remain at earlier stages of implementation. Despite the progress observed, Burundi, Rwanda, and South Sudan continue to face challenges in optimizing the use of antimicrobial agents and in establishing effective systems for monitoring antimicrobial consumption. These findings align with results from the TrACSS country self-assessment[11]. Moreover, ensuring sustainable investment for the implementation of NAP activities remains a critical challenge that all EAC Partner States must address to ensure long-term success in AMR containment.

The WHO Global Strategy to contain AMR recognizes laboratory-based AMR surveillance as a fundamental priority for AMR control strategy development and for assessing the impact of interventions [8]. EAC partner states, except South Sudan, have developed laboratory-based AMR surveillance programs with surveillance sites across these countries. In general, the number and coverage of sites conducting AMR surveillance in the EAC region are low (Table 1). These results align with the findings of the quantitative study conducted by the MAAP group [19] and the reports of the African Society for Laboratory Medicine, which reported a low AMR detection capacity in African countries. [20, 21]. Furthermore, the low clinician demand for bacteriology testing services reported by our expert group and the low coverage of AMR surveillance sites in the EAC region undermine the relevance of laboratory-based surveillance results and could lead to inappropriate strategies or interventions to address the AMR crisis.

In addition, there is limited information about the (often uncontrolled) use of antibiotics country-wide, which makes it difficult to analyze antibiotic prescription patterns [43, 44] and evaluate AMS programs and awareness campaigns [17]. This could be associated with the limited capacity of regulatory bodies to enforce proper use of antibiotics. Moreover, structural barriers such as differing health governance models and language diversity among East African countries could limit the implementation of coordinated and standardized action to contain AMR.

Despite the challenges described, the implementation of stewardship programs in the African region has been shown to improve patient health outcomes and reduce hospitalization costs. [42, 45]

AMR is on the rise globally, with negative public health and economic impacts, and requires a rapid and collective response. Based on our SWOT analysis, we recommend strengthening the existing network between Ministries of Health in the EAC Partner States and developing a regional One Health AMR strategy. Existing regional laboratory networks, such as the East African Community Regional Network of Public Health Reference Laboratories for Communicable Diseases, could be used as a platform for integration into the regional response to AMR.

Some specific recommendations are as follows:

- To connect the EAC partner states with a national multisectoral coordination group for the fight against AMR and create a regional AMR One Health coordination group.
- To enhance, expand and connect the existing laboratory quality assurance and standardization programs for AMR detection to cover the entire region.
- To improve, integrate and expand the different training and e-learning platforms for AMR, AMS and IPC.
- To increase the coverage of the AMR and antimicrobial use surveillance sites in the EAC region to obtain representative regional results, for example, by making use of the EAC mobile laboratories.
- To strengthen the regional supply chain of essential antibiotics and laboratory-diagnostic materials.
- National and regional financial strategies to support AMR activities, including bidding together as a consortium for international funding.

In addition to our primary data focusing on AMR laboratory-based surveillance, it is important to consider other regional initiatives that contribute to AMR mitigation. These include vaccination programs, which help reduce the infectious disease burden and, consequently, the use of antibiotics; Water, Sanitation, and Hygiene (WASH) initiatives, which play a critical role in infection prevention and control; and antimicrobial use (AMU) and stewardship programs. To effectively address AMR, it is crucial that these efforts not remain confined to the human health sector but also involve the animal and environmental health sectors through a coordinated One Health approach [46]. This integrated perspective is essential for tackling the interconnected drivers of AMR and ensuring sustainable, cross-sectoral solutions across the region.

Several global and regional instruments can support this approach. The WHO Implementation Handbook for National Action Plans on Antimicrobial Resistance [22] provides practical tools to achieve the objectives of the Global Action Plan on AMR in alignment with each EAC Partner State’s AMR-NAP. Additionally, the Africa CDC Framework for Antimicrobial Resistance [15], the Antimicrobial Resistance Surveillance Guidance for the African Region [16], and the qualitative baseline assessment conducted in the present study could collectively inform the development of a coordinated EAC regional strategy to combat AMR.

## Conclusion

AMR is a complex problem that puts health systems and economies at risk, especially in developing countries. The Est Africa region has embarked on actions to implement NAPs to control AMR. Despite these efforts, the region faces significant challenges that need to be addressed to counteract this global crisis. Tackling the problem jointly by making use of existing intergovernmental organizations, such as the EAC Health network, and adopting a One Health approach is a way of making the most of existing resources, especially in low- or middle-income countries. The development of a regional AMR One Health Strategy is essential for this endeavor.

## Supplementary Information

Supplementary Material 1

## Data Availability

The datasets used and/or analyzed during the current study are available from the corresponding author upon reasonable request.

## Abbreviations

AMR: Antimicrobial resistance
AMS: Antimicrobial stewardship
EAC: East African community
GLASS: Global antimicrobial resistance surveillance system
IPC: Infection prevention and control
LMICs: Low- and middle-income countries
NAP: National action plan
WHO: World Health Organization

## Data availability

The datasets used and/or analyzed during the current study are available from the corresponding author upon reasonable request.
